# Targeting Epstein–Barr virus is a viable therapeutic strategy in multiple sclerosis: Commentary

**DOI:** 10.1177/13524585251409951

**Published:** 2026-01-14

**Authors:** Anna Karin Hedström

**Affiliations:** Department of Clinical Neuroscience, Karolinska Institutet, Stockholm, Sweden; Department of Neurology, Karolinska University Hospital, Stockholm, Sweden

The two contributions in this Controversy address whether Epstein–Barr virus (EBV) has become a realistic therapeutic target in multiple sclerosis (MS). Steinman argues that recent epidemiological and mechanistic advances have moved the field to a point where targeting EBV could potentially transform MS treatment. Hellings, while acknowledging EBV as the strongest environmental risk factor for MS, cautions that translating these insights into effective therapies for established disease remains far from straightforward.

Recent evidence linking EBV to MS has reached a new level of coherence, providing context for both Steinman’s optimism and Helling’s reservations. The US military cohort showed that EBV infection precedes MS in almost every case and coincides with early signs of neuroaxonal injury.^1^ Molecular mimicry studies have added a mechanistic dimension, showing that somatically mutated antibodies in the cerebrospinal fluid of MS patients target EBNA1 and cross-react with CNS molecules such as GlialCAM, CRYAB and ANO2.^2,3^ These discoveries offer a biologically plausible explanation for how EBV infection, particularly in individuals with susceptible HLA backgrounds, could initiate or amplify CNS-directed autoimmunity.

Against this background, Steinman outlines a broad therapeutic landscape that includes EBV vaccines now entering clinical evaluation, antiviral agents, engineered T- and B-cell-directed therapies, and antigen-specific immune interventions. Conceptually, these approaches appeal because they target upstream drivers of disease rather than its downstream inflammatory consequences. If EBV is indeed fundamental to MS pathogenesis, intervening at this level could achieve effects beyond those of current disease-modifying therapies.

Hellings, however, emphasizes several considerations that temper this optimistic view. EBV’s ability to establish lifelong latency through extensive immune evasion may limit the feasibility of clearing infected cells. Moreover, the same molecular mimicry that supports a causal role for EBV raises the possibility that enhancing antiviral responses could intensify autoreactivity against CNS antigens. Vaccine development has historically struggled to induce sterilizing immunity, and available antivirals lack sufficient specificity for long-term use. And although modern approaches such as mRNA vaccines and EBV-specific T-cell therapies have entered early-phase trials, none has yet shown convincing disease-modifying benefit in established MS.

Taken together, these perspectives suggest that the therapeutic potential of EBV-directed strategies is likely to be stage-dependent. For prevention, an effective EBV vaccine remains an attractive long-term goal, supported by strong causal evidence.^4^ In individuals with established MS, however, EBV-directed approaches remain exploratory, and it is unclear to what extent EBV-related processes continue to drive disease activity after onset.

In sum, accumulating evidence strongly supports EBV-related prevention and continued exploration of EBV-targeted therapies.^5^ Nevertheless, the biological resilience of the virus, risks of amplifying autoreactivity, and the limited clinical experience argue against premature claims of therapeutic viability for established MS. Whether EBV-directed interventions will ultimately benefit individuals with established disease remains uncertain and will depend on evidence generated by future, well-designed clinical trials.
